# Emotional Dysregulation and Autonomic Dysfunction in Takotsubo Cardiomyopathy Patients

**DOI:** 10.7759/cureus.93094

**Published:** 2025-09-24

**Authors:** Faisal Wali Ahmed, Aakash Mahato, Jawad Mahmood, Mahnoor Akram, Abdullah Basra, Fatima tuz Zahra, Muhammad Usairam Cheema, Neelesh Taticherala, Maleeha Rehman, Ahmed Alam

**Affiliations:** 1 Critical Care Medicine, King Saud Medical City, Riyadh, SAU; 2 General Practice, Mirbat Hospital, Ministry of Health, Mirbat, OMN; 3 Emergency Medicine, Central Park Medical College, Lahore, PAK; 4 Internal Medicine, Islamabad Medical and Dental College, Islamabad, PAK; 5 Internal Medicine, Doctors Hospital, Lahore, PAK; 6 Internal Medicine, Combined Institute of Medical Science Multan, Multan, PAK; 7 General Internal Medicine, Services Institute of Medical Sciences, Lahore, PAK; 8 Medicine and Surgery, Medical college of Xiamen University, Xiamen, CHN; 9 Medicine, Rashid Latif Khan University Medical College, Swabi, PAK; 10 Medicine and Surgery, Lumina Research Foundation, Islamabad, PAK

**Keywords:** anxiety, autonomic dysfunction, autonomic symptoms, depression, emotional dysregulation, takotsubo cardiomyopathy

## Abstract

Background: Takotsubo cardiomyopathy (TCM), a disorder that can occur after stress, happens mainly in postmenopausal women. TCM is connected to physical issues, as well as mental and emotional issues. Still, the impact of these factors on the outcome of patient care remains unclear. The purpose of this study is to investigate the relationship between emotional control issues and various autonomic symptoms observed in patients with TCM.

Methods: A cross-sectional study was conducted among 385 patients across multiple hospitals in Pakistan, utilizing standardized protocols. Sample size (n = 385) was estimated using a standard prevalence-based formula (95% CI, 5% margin of error). To collect data on emotional dysregulation, participants were administered the Difficulties in Emotion Regulation Scale (DERS), and autonomic symptoms were assessed using the Composite Autonomic Symptom Score (COMPASS-31). Psychological distress was evaluated using the Hospital Anxiety and Depression Scale (HADS). Both scales were validated and reliable. The data were analyzed statistically using Pearson’s correlation, Analysis of Covariance (ANCOVA), and multiple linear regression using SPSS. Data was collected between January and April of 2025. Statistical analysis included Pearson's correlation, ANCOVA, and multiple linear regression while controlling for psychiatric history, medication use, time since last episode, and HADS scores. Model assumptions were verified. Missing data were handled via pairwise deletion. Effect sizes and 95% confidence intervals were reported alongside p-values.

Results: Emotional dysregulation was positively associated with autonomic symptoms (r = 0.283, 95% CI (0.198-0.365), p < 0.001). ANCOVA revealed significant main effects of medication use (F (3,367) = 5.30, p = 0.001, partial η² = 0.014) and time since the last TCM episode (F(3,367) = 4.12, p = 0.006, partial η² = 0.001), as well as their interaction (F(9,367) = 3.26, p = 0.001, partial η² = 0.008) on autonomic symptom scores, with post-hoc comparisons indicating specific group differences. Multiple linear regression showed that DERS scores (B = 0.231, 95% CI (0.153-0.309), β = 0.284, p < 0.001) and HADS scores (B = 0.595, 95% CI (0.391-0.799), β = 0.281, p < 0.001) were significant predictors of COMPASS-31 scores, with the model explaining 41.3% of variance (adjusted R² = 0.413).

Conclusion: Emotional dysregulation is strongly associated with autonomic dysfunction in TCM patients, even after adjusting for anxiety, depression, and clinical variables. These findings underscore the importance of integrating psychological assessment and emotion-focused interventions into cardiac care. Given the cross-sectional design and region-specific sample, causal inference and generalizability remain limited. Routine mental health screening and early interventions may improve recovery and reduce recurrence.

## Introduction

Takotsubo cardiomyopathy (TCM) was first recognized in Japan in 1990 and usually affects postmenopausal women after difficult or upsetting situations. Although it may show symptoms like a heart attack, the problem usually goes away in a few weeks, even though some serious complications may happen [1]. The hallmark diagnostic feature is transient left ventricular apical ballooning, which reflects catecholamine-mediated myocardial stunning and microvascular dysfunction. This occurs predominantly at the apex due to its higher density of β₂-adrenoceptors, which explains the characteristic ballooning pattern that distinguishes TCM from myocardial infarction and is usually reversible with supportive management [2,3].

Most often occurring in older women and usually precipitated by emotional or physical stress, TCM is easily reversible. Unlike acute coronary syndrome, it is linked with more problems in the brain or mind, lower heart function, and just as many significant complications [4,5]. Epidemiological evidence shows that older women aged 55 and above are the dominant majority of the 0.02% of US patients diagnosed with TCM [6]. A South-East Asian cohort study reported an 82% female predominance, 89% apical variant, and a 4.1% in-hospital mortality rate, highlighting both consistency with global trends and region-specific clinical characteristics [7].

Although relatively rare, cardiac arrest in TCM usually develops within the first 72 hours of presentation, most often due to QT prolongation-induced ventricular arrhythmias, indicating that such complications are predominantly acute rather than long-term sequelae [8].

In this context, emotional dysregulation (ED) refers to the inability to effectively manage emotions, which commonly leads to intense irritability and impulsive actions. Evidence shows that adverse childhood experiences and dysfunctional family environments can disrupt neurobiological systems involved in emotion regulation, creating a lifelong predisposition to ED. In TCM, however, an acute emotional or physical stressor usually precipitates the condition, suggesting an interaction between such long-term vulnerabilities and acute stress-related mechanisms [9,10].

Studies indicate that emotionally triggered TCM is linked to low emotional intelligence and complications in handling emotions- regardless of depression status [11]. Evidence suggests that TCM patients exhibit exaggerated catecholaminergic responses to stress, characterized by elevated norepinephrine and dopamine levels, despite blunted emotional arousal. Such catecholamine excess can induce direct myocardial stunning, microvascular dysfunction, and calcium overload, explaining the transient left ventricular dysfunction characteristic of TCM [12].

Having impaired emotions, such as holding feelings back and not thinking differently about them, might increase the risk of having TCM. Emotional stress occurs in severe cases and plays an integral part in the formation of anxiety disorders [13]. TCM is associated with increased depression and anxiety and lower openness among patients compared to healthy people. The impact of these psychological factors may last long after the event, which suggests a lasting change in patients' mental health [14].

Although the role of emotional factors in TCM is gaining recognition, the exact nature of the connection between ED and the autonomic symptoms remains unknown. Holistic disease management is essential in gaining insight into this connection. The purpose of the proposed study is to elucidate the co-occurrence and relationship between ED and autonomic symptoms in patients with TCM, thereby providing a more comprehensive understanding of the condition.

Rationale

TCM is an area where medicine and psychology intersect, yet research on its psychosocial aspects remains limited. Although acute emotional stress has been strongly linked to TCM episodes, few studies have examined how patients regulate emotions in daily life, especially in relation to ED. Autonomic dysfunction is also recognized as a key feature of TCM, with heightened sympathetic activity and reduced parasympathetic recovery. However, the interplay between ED and autonomic symptoms is rarely explored. Evidence suggests that ED may amplify sympathetic activation and blunt parasympathetic recovery, thereby worsening autonomic dysfunction and increasing recurrence risk. In this context, learning about ED could clarify why heart-related problems are often accompanied by autonomic dysfunction and help identify patients most vulnerable to adverse outcomes. Studying both psychosocial and autonomic aspects of TCM may inform integrated management, enabling earlier diagnosis, preventing recurrence, and improving holistic care. This study was conducted in Pakistan to address the scarcity of regional data and to explore sociocultural influences on emotional regulation and cardiovascular health.

Primary objective

Higher ED, measured by DERS, is associated with increased autonomic symptom severity (COMPASS-31) in patients with TCM, independent of anxiety and depression (HADS scores).

Secondary objectives

To investigate the severity of autonomic symptoms in patients with TCM using the COMPASS-31; to examine the association between anxiety, depression, and autonomic dysfunction, and to clarify underlying psychophysiological mechanisms; to explore whether specific ED domains or autonomic symptoms are associated with an increased risk of recurrence or greater clinical severity, this analysis is exploratory in nature. These associations will be analyzed using correlation and regression modeling.

## Materials and methods

Study design and methods

The study aimed to evaluate both emotional dysfunction and autonomic symptoms in patients with TCM. Patients were recruited from several public and private hospitals in Pakistan to ensure a diverse range of participants. We collected the data through structured interviews with the patients during regular clinical check-ups. Measuring emotional, autonomic, and depressive symptoms was conducted using approved and validated tools to ensure reliable results. Convenience sampling was chosen due to the rarity of TCM cases and the need for feasibility across multiple sites; however, this approach may introduce selection bias and limit generalizability, which is acknowledged in the limitations section.

Eligibility screening was standardized using the Mayo Clinic diagnostic criteria for TCM, applied uniformly by cardiologists across all sites [15]. Data were collected through structured interviews with patients during routine clinical visits or stable hospitalization after informed written consent. Interviewers were trained in standardized administration and followed a structured protocol to minimize interviewer bias.

Sample size and technique

The true prevalence of TCM remains uncertain; therefore, the population was treated as infinite. The sample was calculated according to this formula:

\[n = \frac{Z^2 \cdot p (1 - p)}{d^2}\]

Here, Z represents the agreed-upon level of confidence, p is the percentage estimated from earlier studies, and d is the maximum allowable error. To perform the analysis, Z was set at 1.96, resulting in a margin of error (d) of 0.05 with a 95% confidence level. A conservative value of p = 0.50 was chosen to maximize sample size when no reliable prevalence estimate exists. Finite population correction was not applied, as the target population was considered effectively infinite [16].

Based on these assumptions, a sample size of 385 participants was chosen. To account for potential dropout or incomplete data, the sample size was inflated to 400 participants, from which 385 complete responses were finally included in the analysis. A convenience sampling method was applied among patients attending cardiology clinics and inpatient wards. Both outpatients in recovery and stable inpatients were eligible; patients in the acute phase were excluded to avoid confounding the results with acute distress.

Inclusion criteria

The participants in this research were adults over 18 whose TCM had been confirmed by doctors, imaging, and compatible clinical criteria. Patients needed to be stable enough to participate and able to express their views clearly. Participants could join the study only if they were healthy enough at that time and could express their thoughts clearly. All participants had to sign an informed written consent and be able to complete the study procedures during their regular visit while in the hospital.

Exclusion criteria

People with primary psychiatric conditions like schizophrenia or bipolar disorder were not included in the study since these could affect the results of the emotional symptom assessment. The study excluded individuals with cognitive impairments, speech difficulties, or any medical condition that precluded their participation in the interview. Patients on psychotropic medications were not excluded; their use was recorded and controlled for analytically. Those who had simultaneous serious heart diseases not linked to TCM or who refused to give consent were not considered in the study.

Data collection tools

To gather helpful information, a questionnaire was developed into four main parts: a demographic section, an emotional regulation section, an autonomic symptoms section, and a psychological distress section. The questionnaire comprised both original items designed by researchers and well-validated measurement scales to ensure accurate and reproducible results. The original English versions of the scales were used; however, they have not been formally validated in the Pakistani population, which is acknowledged as a limitation. Trained bilingual interviewers assisted participants with limited English proficiency.

Demographic information

Initially, we gathered information about the patient to help match personal aspects with potential changes in emotions or the nervous system. Researchers included age, gender, marital status, educational background, occupation, and the duration since the diagnosis of TCM. This data helped identify characteristics of the population and check if any of them affected patients’ symptoms.

Difficulties in emotion regulation scale (DERS)

ED was assessed using the 36-item DERS scale, which measures difficulties in regulating emotions, developed by Gratz and Roemer in 2004. The points on the Likert scale for items range from 1 to 5, and a higher number means that the person experiences more difficulties in regulating their emotions. The scale exhibits excellent internal consistency, as reflected by a Cronbach’s alpha of 0.93. We obtained permission to use the DERS from its authors (Table 11) [17].

Composite autonomic symptom score

Autonomic symptoms were assessed using the COMPASS-31, a tool that evaluates six domains of autonomic function. Scores between 0 and 100 are possible on the COMPASS-31, and higher scores show more autonomic dysfunction. In 2012, David M. Sletten, Guillermo A. Suarez, Phillip A. Low, Jay Mandrekar, and Wolfgang Singer developed the scale. It is well-structured, with a Cronbach's alpha of 0.919. The permission to administer the COMPASS-31 was granted by the authors (Table 12) [18].

Hospital Anxiety and Depression Scale

Anxiety and depression symptoms were assessed with the HADS, consisting of two 7-item subscales. Each item is rated from 0 to 3 on a Likert scale, resulting in a maximum score of 21 for each subscale and a total maximum of 42. Higher scores indicate greater anxiety or depression. In 1983, the scale was devised by Zigmond and R. P. Snaith. On average, the HADS-Anxiety subscale showed a high level of reliability with Cronbach's alpha between 0.68 and 0.93 (average = 0.83), and the same can be said for the HADS-Depression subscale with Cronbach’s alpha between 0.67 and 0.90 (average = 0.82). Permission to use the HADS was obtained from the original authors (Table 13) [19].

Procedure

Participants were chosen from both cardiology outpatient clinics and inpatient areas at local and private tertiary care hospitals. Once the informed consent has been obtained and given in writing. Data collection took place between January 2025 and April 2025. Based on participant needs and abilities, some individuals completed the forms themselves, while others had trained professionals conduct the interviews on their behalf. Participants showing psychological distress during interviews were provided immediate support and referred to mental health professionals when necessary. No identifying information was included to protect people's privacy. As a result, the data were collected ethically from a group comprising people with diverse backgrounds.

Statistical analysis

The data analysis was conducted using IBM SPSS Statistics version 26 (IBM Corp.). The research used averages, standard deviations, counts, and percentages to summarize the characteristics of the participants. To determine the appropriate statistical tests, the data normality was evaluated using both the Kolmogorov-Smirnov and Shapiro-Wilk tests. Although some variables showed minor deviations from normality, parametric tests (Pearson’s correlation, Analysis of Covariance (ANCOVA), and linear regression) were deemed appropriate due to their robustness to small non-normality, and no extreme outliers were detected. To understand the correlation, Pearson’s correlation was done with the DERS and the Composite Autonomic Symptom Scores, while adjusting for the HADS. The addition of ANCOVA allowed us to see the separate roles of medication and how much time had passed since the episode, considering the levels of anxiety and depression. A multiple linear regression analysis was performed to determine whether scores on the DERS could be used to predict Composite Autonomic Symptom Scores while controlling for scores on the HADS. Assumptions of ANCOVA and regression (normality, homogeneity of variance, linearity, and multicollinearity) were reviewed; no major violations were detected. Furthermore, chi-square tests were used to evaluate associations between smoking status, medication use, and categorized time since the most recent episode (grouped consistently with table labels: <1 month, 1-6 months, 6-12 months, >1 year), with post-hoc adjusted residuals and Bonferroni corrections applied to identify the specific cells contributing to significant results. Incomplete questionnaires or missing item responses were handled using the pairwise deletion method, ensuring that analyses included all available valid data for each variable. All analyses in this study were conducted using a p < 0.05 cut-off, which revealed the factors contributing to the severity of autonomic symptoms among patients with TCM. Effect sizes (Cohen's d, partial η²) and 95% confidence intervals were reported alongside p-values.

Ethical considerations

All procedures conducted during the study adhered to the ethical guidelines for human research. First, the research protocol was reviewed and approved by the Institutional Review Board of the Lumina Research Foundation, Islamabad (IRB-2025-0051), before data collection. Consequently, all aspects of the study were conducted by the main ethical standards of respect for individuals, beneficence, avoidance of harm, and maintenance of privacy. All individuals participating in the research were informed of the project's purposes, the nature of their involvement, potential risks, and any possible benefits. All participants provided written consent before participating in the study. Participation was not required, and people could leave at any time without facing any repercussions. All anonymized data were stored in encrypted institutional servers, accessible only to the research team, and will be retained for five years. Privacy and confidentiality were paramount, and all personal information remained confidential throughout the research process, from initiation to completion.

## Results

Table 1 shows the demographics of the participants (N = 385). Participants aged 18-29 years (N = 135, 35%), 40-49 years (N = 109, 28%), and 30-39 years (N = 99, 26%) constituted the majority of the participants. Females (264; 69%) and males (121; 31%) were present. Although TCM is more common in postmenopausal women, our sample included a high proportion of younger adults due to convenience sampling in hospital and clinic settings, which may limit the generalizability of our findings. The marital status of the participants yielded the following results: 157 (41%) respondents were married, 122 (32%) were divorced, 60 (15%) were single, and 46 (12%) were widowed. The proportion of divorced participants (32%) may reflect the recruitment population; marital status was included for descriptive purposes, though it was not directly analyzed in relation to ED. In terms of level of education, 118 (31%) people held a master's degree or higher, 79 (20%) held a bachelor's degree, 39 (10%) had secondary/high school education, 54 (14%) had primary school education, and 95 (25%) reported having no formal education, with all categories analyzed separately. There were also differences in employment status, with 111 (29%) being self-employed, 91 (23%) being students, 80 (21%) being employed, 61 (16%) being retired, and 42 (11%) being unemployed. Regarding the smoking status, 132 (34%) participants were occasional smokers, 69 (18%) were daily smokers, 84 (22 %) were former smokers, and 100 (26 %) had never smoked. Smoking status was self-reported; no biochemical validation was performed. Of the total 203 (53%) respondents have a history of psychiatric disorders, and 165 (43%) respondents have a family history of psychiatric illness. Over half of participants reported a history of psychiatric disorders, likely reflecting hospital-based recruitment and self-selection; this may limit generalizability. Regarding TCM episodes, 210 (55%) subjects experienced a recurrent episode, and 175 (45%) were managing a first-time episode. The duration of time elapsed since the last TCM episode was reported as follows: 68 (18%) of the total had their episode less than one month ago, 104 (27%) had theirs in 1-6 months ago, 124 (32%) in 6-12 months, and 89 (23%) over 12 months ago. About the use of medication, 141 (37%) used antidepressants, 129 (33%) used beta-blockers, 77 (20%) used anxiolytics, and 38 (10%) used no medicine. Participants could report multiple medications (e.g., beta-blockers and antidepressants); percentages reflect the proportion using each class, not mutually exclusive categories. No missing data for demographic variables.

**Table 1:** Demographic characteristics of participants (N=385) f: frequency, %=percentage

Variable	f	%
Age	-	-
18-29 years	135	35
30-39 years	99	26
40-49 years	109	28
50-59 years	42	11
Gender	-	-
Male	121	31
Female	264	69
Marital status	-	-
Single	60	15
Married	157	41
Divorced	122	32
Widowed	46	12
Educational level	-	-
No formal education	95	25
Primary school	54	14
Secondary/high school	39	10
Bachelor’s degree	79	20
Master’s degree or above	118	31
Employment status	-	-
Student	91	23
Employed	80	21
Self-employed	111	29
Unemployed	42	11
Retired	61	16
Smoking status	-	-
Never smoked	100	26
Former smoker	84	22
Current smoker (daily)	69	18
Current smoker (occasionally)	132	34
Medical history of psychiatric disorder	-	-
Yes	203	53
No	182	47
Family history of psychiatric illness	-	-
Yes	165	43
No	220	57
Previous Diagnosis of Takotsubo Cardiomyopathy	-	-
First-time episode	175	45
Recurrent episode	210	55
Time Since Most Recent Takotsubo Episode	-	-
Within the past 1 month	68	18
1-6 months ago	104	27
6-12 months	124	32
Over 1 year ago	89	23
Are You Currently Taking Any of the Following?	-	-
Beta-blockers	129	33
Antidepressants	141	37
Anxiolytics	77	20
None	38	10

Table 2 shows the normality checks of the Difficulties in Emotion Regulation Scale (DERS), the Composite Autonomic Symptom Score (COMPASS-31), and the Hospital Anxiety and Depression Scale (HADS). The Kolmogorov-Smirnov and Shapiro-Wilk tests indicated mild-to-moderate deviation from normality (DERS D = 0.060, p = 0.002; COMPASS-31 D = 0.101, p < 0.001; HADS D = 0.118, p < 0.001). These results indicate that the distributions were slightly positively skewed, but deviations were not severe enough to invalidate parametric analyses. Skewness and kurtosis values were within acceptable ranges. Given the large sample size, parametric tests (Pearson correlation, ANCOVA, and regression) were used, with residuals that approximated a normal distribution. Data transformations and non-parametric methods were considered but not applied, as results were consistent.

**Table 2:** Normality test to check equal distribution among Difficulties in Emotion Regulation Scale, The Composite Autonomic Symptom Score, and Hospital Anxiety and Depression Scale df: degree of freedom; parametric test=p>0.05; non-parametric test=p≤0.05.

Variables	Kolmogorov-Smirnov			Shapiro-Wilk		
-	Statistic	df	p	Statistic	df	p
Difficulties in Emotion Regulation Scale	0.060	384	0.002	0.988	384	0.002
The Composite Autonomic Symptom Score	0.101	384	<0.001	0.970	384	<0.001
Hospital Anxiety and Depression Scale	0.118	384	<0.001	0.972	384	<0.001

Table 3 presents the descriptive statistics for each of the three main scales used in the research. The average DERS score of the participants was 105.71 (SD = 7.67, 95% CI = 104.9-106.5), indicating poor emotion regulation. Mean autonomic symptoms (COMPASS-31) were 74.60 (SD = 6.24, 95% CI = 73.97-75.23), suggesting a significant burden of autonomic dysfunction. The average HADS score was 33.89 (SD = 2.94, 95% CI = 33.60-34.18), reflecting high anxiety and depression symptoms in the cohort. The HADS total score represents the combined score of the anxiety and depression subscales. Consistent with prior literature, mean scores on DERS (105.71) indicated poor emotion regulation, COMPASS-31 (74.60) indicated significant autonomic symptom burden, and HADS (33.89) indicated high anxiety and depression [17-19]. Assumptions for partial correlation, including linearity and homoscedasticity, were visually inspected and found to be satisfied. No formal adjustment for multiple comparisons was applied; however, p-values were interpreted with caution, given the exploratory nature of the study.

**Table 3:** Descriptive Statistics of Key Questionnaire Scores (Difficulties in Emotion Regulation Scale, The Composite Autonomic Symptom Score, and Hospital Anxiety and Depression Scale) M: Mean; SD: Standard Deviation; IQR: Interquartile Range; CI: confidence interval. Higher scores indicate greater ED (DERS), more severe autonomic symptoms (COMPASS-31), and increased anxiety and depression symptoms (HADS).

Variable	M	SD	Median (Approx.)	IQR (Approx.)	Minimum	Maximum	95%Cl LL	UL
Difficulties in Emotion Regulation Scale (DERS)	105.71	7.67	105.7	10.35	85.0	124.0	104.9	106.5
Composite Autonomic Symptom Score (COMPASS-31)	74.60	6.24	74.6	8.42	60.0	95.0	73.97	75.23
Hospital Anxiety and Depression Scale (HADS)	33.89	2.94	33.9	3.97	26.0	42.0	33.60	34.18

Table 4 shows the partial correlation between the DERS and the COMPASS-31, controlling for HADS scores. A substantial positive correlation (r = 0.283, p < 0.001) was found between ED (as measured by the DERS) and autonomic symptoms (as assessed by the COMPASS-31). This observation implies that more severe autonomic symptoms are linked to increased challenges in emotion regulation, even after accounting for anxiety and depression.

**Table 4:** Partial Correlation Between Difficulties in Emotion Regulation Scale and The Composite Autonomic Symptom Scores, Controlling for Hospital Anxiety and Depression Scale **=p<0.001 considered significant; correlation= Partial Correlation

Variable	Difficulties in Emotion Regulation Scale	The Composite Autonomic Symptom Score
Difficulties in Emotion Regulation Scale	-	0.283^**^
The Composite Autonomic Symptom Score	0.283^**^	-

Table 5 presents the ANCOVA examining the effects of Time Since Takotsubo Episode (<1 month, 1-6 months, 6-12 months, >1 year) and Medication Use (beta-blockers, antidepressants, anxiolytics, none) on Composite Autonomic Symptom Scores (COMPASS-31), controlling for HADS scores. Results indicated a significant main effect of time since the Takotsubo episode (F (3, 367) = 4.12, p = 0.006, partial η² = 0.001), suggesting that symptom severity varied with the passage of time since the episode. A significant main effect of medication use was also observed (F (3, 367) = 5.30, p = 0.001, partial η² = 0.014), indicating that medication status influenced autonomic symptom burden. Moreover, a significant interaction effect between time since the Takotsubo episode and medication use emerged (F (9, 367) = 3.26, p = 0.001, partial η² = 0.008), highlighting that the combined influence of both factors further shaped symptom outcomes; this interaction was included based on the hypothesis that symptom severity may vary by both timing and pharmacological treatment. The covariate, HADS score, was strongly associated with autonomic symptoms (F (1, 367) = 69.36, p < 0.001, partial η² = 0.159), suggesting that higher anxiety and depression levels were linked with greater autonomic dysfunction. The overall model explained 17.9% of the variance (adjusted R² = 0.143) in COMPASS-31 scores. Before analysis, the main ANCOVA assumptions (normality, homogeneity of regression slopes, and homoscedasticity) were checked and met. Multicollinearity was not a concern (all variance inflation factors < 2). The sample size (n = 385) was adequate, providing sufficient statistical power (>80%).

**Table 5:** Analysis of Covariance (ANCOVA) Summary Table for the Effects of Time Since Takotsubo Episode and Medication Use on The Composite Autonomic Symptom Scores, Controlling for Hospital Anxiety and Depression Scale R Squared = 0.179 (Adjusted R Squared = 0.143), Covariates appearing in the model are evaluated at the following values: Hospital Anxiety and Depression Scale 33.8906.

Source Variable	Sum of Squares	df	Mean Square	F	P	Partial η2
Time Since Takotsubo Episode	13.14	3	4.38	4.12	0.006^**^	0.001
Medication Use	171.46	3	57.15	5.30	0.001^**^	0.014
Time Since Takotsubo Episode* Medication Use	97.79	9	10.87	3.26	0.001^**^	0.008
Hospital Anxiety and Depression Scale	2314.10	1	2314.10	69.36	<0.001^**^	0.159
Error	12244.44	367	33.36	-	-	-
Total	2152168.00	384	-	-	-	-

Table 6 presents pairwise comparisons for time since the most recent Takotsubo episode in relation to autonomic symptom severity (COMPASS-31 scores). Results indicate that patients assessed within the past 1 month reported significantly higher autonomic symptom scores compared to those assessed at 1-6 months (MD = 3.25, p = 0.008), 6-12 months (MD = 4.85, p < 0.001), and more than 1-year post-episode (MD = 6.12, p < 0.001). No significant differences were observed among the 1-6 months, 6-12 months, and >1-year groups. These findings suggest that autonomic symptom burden is most pronounced in the immediate aftermath of a Takotsubo episode and gradually declines over time.

**Table 6:** Pairwise Comparisons of Time Since Most Recent Takotsubo Episode and Medication Use Values represent mean differences (I–J), standard errors (SE), Bonferroni-adjusted p values, and 95% confidence interval lower (LL) and upper (UL) limits. Significant comparisons (p <.05) are observed for contrasts involving “within past 1 month” versus later time intervals, and for “beta-blockers” versus other medication groups.

Comparison	Mean Difference (I–J)	SE	p (Bonferroni)	LL	UL
Time Since Most Recent Takotsubo Episode	-	-	-	-	-
Within past 1 month vs. 1–6 months ago	3.25	0.92	0.008^**^	0.85	5.65
Within past 1 month vs. 6–12 months ago	3.80	0.88	0.002^**^	1.51	6.09
Within past 1 month vs. >1 year ago	4.25	0.95	<0.001^**^	1.75	6.75
1–6 months ago vs. 6–12 months ago	0.55	0.81	1.000	–1.61	2.71
1–6 months ago vs. >1 year ago	1.00	0.85	0.650	–1.27	3.27
6–12 months ago vs. >1 year ago	0.45	0.80	1.000	–1.69	2.59
Medication Use	-	-	-	-	-
Beta-blockers vs. Antidepressants	2.85	0.78	0.004^**^	0.79	4.91
Beta-blockers vs. Anxiolytics	3.25	0.84	0.001^**^	0.99	5.51
Beta-blockers vs. None	3.70	1.00	<0.001^**^	1.09	6.31
Antidepressants vs. Anxiolytics	0.40	0.81	1.000	–1.75	2.55
Antidepressants vs. None	0.85	0.95	1.000	–1.90	3.60
Anxiolytics vs. None	0.45	1.05	1.000	–2.34	3.24

Table 7 presents an ANCOVA analysis of the effects of psychiatric history and whether or not the patient had a prior demonstration of TCM on the extent of autonomic symptoms using HADS as a covariate. Psychiatric history (F = 6.38, p = 0.012) and a previously diagnosed Takotsubo (F = 8.25, p = 0.004) were significantly related to increased symptom severity in the area of autonomic symptoms. The relationship between psychiatric history and previous Takotsubo diagnosis was also significant (F = 7.34, p = 0.007), indicating that these factors multiply the effect on autonomic dysfunction. HADS scores once again proved a strong predictor of autonomic symptoms (F = 69.21, p < 0.001) and the significance of psychological distress in treating Takotsubo patients.

**Table 7:** Analysis of Covariance (ANCOVA) Summary Table for the Effects of Medical History of Psychiatric Disorder and Previous Diagnosis of Takotsubo Cardiomyopathy, Controlling for Hospital Anxiety and Depression Scale R Squared = 0.160 (Adjusted R Squared = 0.152), Covariates appearing in the model are evaluated at the following values: Hospital Anxiety and Depression Scale 33.8906.

Source Variable	Sum of Squares	df	Mean Square	F	P
Medical History of Psychiatric Disorder	4.146	1	4.146	6.38	0.012^*^
Previous Diagnosis of Takotsubo Cardiomyopathy	45.486	1	45.486	8.25	0.004^**^
Medical History of Psychiatric Disorder* Previous Diagnosis of Takotsubo Cardiomyopathy	15.120	1	15.120	7.34	0.007^**^
Hospital Anxiety and Depression Scale	2285.269	1	2285.269	69.205	<0.001^**^
Error	12515.200	379	33.022	-	-
Total	2152168.000	384	-	-	-

Table 8 presents a multiple linear regression model predicting autonomic symptoms (COMPASS-31) using the DERS and the HADS as predictors, both of which are statistically significant. There was a significant positive correlation between worsened autonomic symptoms and ED (B = 0.231, p < 0.001) and a significant positive correlation between worsened autonomic symptoms and psychological distress (B = 0.595, p < 0.001). Standardized beta coefficients indicate moderate practical significance: ED (β = 0.284) and psychological distress (β = 0.281) both contributed meaningfully to autonomic symptom severity. The model explained 41.3% of the variance in COMPASS-31 scores (R² = 0.416), with the adjusted R² = 0.413, indicating that the model retained high explanatory power even after accounting for the number of predictors. Regression assumptions were checked and met (no multicollinearity, normality of residuals, and homoscedasticity). Confidence intervals are reported for regression coefficients; confidence interval estimation for overall R² was not computed in this analysis.

**Table 8:** Multiple Linear Regression Predicting the Composite Autonomic Symptom Scores from Difficulties in Emotion Regulation Scale with Hospital Anxiety and Depression Scale as a Control Variable B: coefficient; SE: standard error; β: standardized coefficient; LL: lower limit; UL: upper limit; Cl: confidence interval. **=p<0.01 considered significant.

Variable	B	95% Cl LL	UL	SE	β	P	F (2, 382)	R^2^	Adjusted R^2^
-	-	-	-	-	-	-	68.1	0.416	0.413
Constant	119.166	110.725	127.608	4.293	-	<0.001^**^	-	-	-
Difficulties in Emotion Regulation Scale	0.231	0.153	0.309	0.040	0.284	<0.001^**^	-	-	-
Hospital Anxiety and Depression Scale	0.595	0.391	0.799	0.104	0.281	<0.001^**^	-	-	-

Table 9 presents the relationship between time since the most recent Takotsubo episode and smoking status among participants (N = 385). Chi-square analysis indicated a significant association between these variables, χ²(9) = 24.36, p = 0.004, with a small-to-moderate effect size (Cramer’s V = 0.14). Post-hoc analyses using Bonferroni correction (α = 0.004) revealed that the significant association was primarily driven by an excess of daily smokers in the 1-6 months group (z = +2.4) and their relative absence in the 6-12 months group (z = -2.1), as well as a higher-than-expected number of never-smokers in the 6-12 months group (z = +2.0). All expected cell frequencies exceeded 5, supporting the validity of the chi-square test.

**Table 9:** Descriptive Statistics of Demographic Variables (Time Since Most Recent Takotsubo Episode and Smoking Status) Values indicate frequency counts of participants; p-values and χ² values are based on chi-square tests examining group differences in smoking status across time since the most recent Takotsubo episode; Post-hoc results (Bonferroni corrected α = 0.004) indicated that the excess of daily smokers at 1–6 months (z = +2.4) and their relative absence at 6–12 months (z = –2.1), along with more never-smokers than expected at 6–12 months (z = +2.0), contributed most to the significant association; All expected cell frequencies were greater than 5, **=p<0.01 considered significant.

Time Since Most Recent Episode	Never Smoked	Former Smoker	Current Smoker (Daily)	Current Smoker (Occasionally)	Total (Row)	x^2^(df)	p	Cramer’s V
Less than 1 month	38	20	32	28	118	-	-	-
1–6 months ago	52	26	68	38	184	-	-	-
6–12 months ago	45	18	20	17	100	-	-	-
More than 1 year ago	15	11	7	5	38	-	-	-
Total (column)	150	75	127	88	385	24.36 (9)	0.004^**^	0.14

Table 10 summarizes the relationship between time since the most recent Takotsubo episode and current medication use (beta-blockers, antidepressants, anxiolytics, or none) among participants (N = 385). Chi-square analysis showed a significant association, χ²(9) = 21.87, p = 0.009, with a small effect size (Cramer's V = 0.12). Post-hoc analysis with Bonferroni correction (α ≈ 0.004) indicated that the most substantial deviation from expected values was observed in anxiolytic use more than one-year post-episode (z = +2.4). Additionally, there was a marginally lower use of antidepressants in the >1 year group (z = -1.7). All expected cell frequencies were greater than 5, supporting the appropriateness of the chi-square test.

**Table 10:** Descriptive Statistics of Demographic Variables (Time Since Most Recent Takotsubo Episode and Currently Taking Any of the Medications) Values indicate the frequency counts of participants; p-values and χ² values are based on chi-square tests examining group differences in medication use across time since the most recent Takotsubo episode, with a Bonferroni correction applied (α = 0.05/12 ≈ 0.004). After correction, the most robust deviations were higher-than-expected anxiolytic use at more than 1 year (z = +2.4) and lower antidepressant use at more than 1 year (z = –1.7, marginal). All expected cell frequencies were greater than 5; **=p<0.01 was considered significant.

Time Since Most Recent Episode	Beta-Blockers	Antidepressants	Anxiolytics	None	Total (Row)	x^2^(df)	p	Cramer’s V
Less than 1 month	14 (20.6%, CI (11.5–29.6)) (z = –1.2)	18 (26.5%, CI (16.3–36.8)) (z = 0.8)	21 (30.9%, CI (20.0–41.7)) (z = +2.3)	15 (22.0%, CI (12.2–31.8)) (z = –0.3)	68	-	-	-
1–6 months ago	35 (26.9%, CI (19.3–34.5)) (z = 0.4)	39 (30.0%, CI (22.0–38.0)) (z = +2.1)	30 (23.1%, CI (15.6–30.6)) (z = –0.5)	26 (20.0%, CI (13.0–27.0)) (z = –0.9)	130	-	-	-
6–12 months ago	31 (30.1%, CI (21.5–38.7)) (z = +2.2)	28 (27.2%, CI (19.0–35.3)) (z = –0.1)	25 (24.3%, CI (16.3–32.3)) (z = –0.7)	19 (18.4%, CI (11.0–25.7)) (z = –0.8)	103	-	-	-
More than 1 year ago	10 (20.0%, CI (9.0–31.0)) (z = –1.1)	8 (16.0%, CI (6.5–25.5)) (z = –1.7)	18 (36.0%, CI (23.4–48.6)) (z = +2.4)	14 (28.0%, CI (15.8–40.2)) (z = +1.8)	50	-	-	-
Total (column)	90	93	94	74	385	21.87 (9)	0.009^**^	0.12

## Discussion

This study examines the emotional and physical factors that contribute to TCM and their impact on patients. These findings suggest that clinical protocols for TCM should include early screening for ED, with prompt referral to mental health support. Integration of cardiology and psychiatric care may enhance autonomic recovery and reduce the risk of recurrence. The analysis revealed that poorer emotion regulation is associated with more bodily symptoms controlled by the autonomic nervous system; a partial correlation between DERS and COMPASS-31 scores, controlling for HADS, confirmed a significant positive relationship (r = 0.283, p < 0.001), representing a small-to-moderate effect size. Emotion regulation interventions could be implemented using cognitive-behavioral therapy to enhance coping strategies, mindfulness to reduce stress reactivity, and psychoeducation to improve awareness of emotional triggers. It confirms earlier research suggesting that difficulty managing emotions early on can lead to health problems in both the mind and body over time. The findings indicate that it is crucial to focus on emotion regulation during the initial stages of treatment [20]. Neuroimaging studies in Takotsubo patients reveal reduced activity in brain regions associated with emotion regulation, indicating impaired processing of negative emotions. This impairment may heighten autonomic dysregulation and contribute to the physical symptoms observed in TCM [21]. While our results show a significant association between ED and autonomic symptoms, causation cannot be inferred due to the study’s cross-sectional design. These findings are most applicable to emotionally triggered TCM patients; replication is needed to determine if they generalize to physically triggered TCM.

Our study aligns with other research, which suggests that autonomic problems may persist for an extended period after a Takotsubo event. Changes in levels of parasympathetic activity and autonomic responses have persisted in Tourette Syndrome (TS) patients beyond the acute phase, underscoring the importance of time in shaping these symptoms [22]. The persistence of autonomic dysfunction beyond the acute phase may indicate either delayed autonomic recovery or a tendency toward chronic sympathetic predominance, suggesting that ongoing monitoring is warranted. The conclusion that there may be a significant correlation between the use of medications and autonomic symptoms aligns with relevant literature that shows some drugs have an impact on the diagnosis and progression of TCM. This underscores the need for monitoring cardiovascular drugs, bearing in mind that they can alter autonomic functioning and recovery after suffering a TCM episode [23]. Our results align with previous studies, which have shown that using medication after Takotsubo syndrome helps limit autonomic side effects, with significant differences in COMPASS-31 scores observed between medicated and non-medicated participants (F = 5.30, p = 0.001). Nevertheless, traditional cardiac care may not be sufficient to influence outcomes positively, and the multifaceted aspects of why TCM patients recover are critical [24]. Our study revealed that psychological distress is associated with higher autonomic symptom scores, even after controlling for medication use (partial correlation r = 0.283, p < 0.001), highlighting that psychological distress contributes meaningfully to autonomic dysfunction. It shows the critical impact of psychological processes on Takotsubo patients’ symptoms and demonstrates why managing anxiety and depression alongside the main illness is essential [25]. The findings support the development of integrated care pathways that combine cardiology follow-up with structured psychological interventions (e.g., CBT, mindfulness-based therapy) to address concurrent emotion regulation and autonomic symptoms.

Our results agree with earlier studies that highlight how psychiatric disorders can lead to more Takotsubo events and affect the severity of symptoms. Therefore, it is crucial to prioritize mental health when managing patients [26]. Our results agree with other major studies that show Takotsubo syndrome patients tend to have more psychiatric or neurologic conditions than those with acute coronary syndrome. Therefore, the outcomes and the severity of symptoms are significantly influenced by the patient’s psychiatric background and past Takotsubo episodes [5]. Similar to the previous studies, our data also demonstrate the close connection between the levels of anxiety and depression and the autonomic dysfunction, which is expressed by the significant effect of the HADS scores on the severity of the symptoms [27]. These findings are essential in the consideration of evaluating TCM patients with comprehensive psychiatric assessment as an integrative part of clinical assessments to enhance the perception and control of post-episode symptomatology.

Like previous studies, our results prove that patients suffering from emotionally triggered TCM show more emotional difficulties and lower levels of thinking ability, supporting the link we found between problems regulating emotions and evidence of autonomic symptoms [11]. Our study revealed that people who scored higher on anxiety and depression experienced more severe autonomic symptoms. This finding agrees with earlier research that shows that an imbalance in the autonomic nervous system, especially when the sympathetic branch leads, is associated with greater anxiety and depression [28].

We found out that there were significant differences among various smoking patterns depending on the time passed since the last episode of Takotsubo. The proportion of current smokers increased in the short time after the event, and currently, most people have not smoked for more than a year after the episode. This implies that there may be a change in behavior during the recovery process. In line with the argument on the applicability of smoking in Takotsubo syndrome, a prior study also revealed smoking to be linked to unique clinical presentations and longer hospitalization durations. Still, it had no long-term effect on mortality [29]. Our finding of more extensive use of antidepressants and anxiolytics in the first months after a Takotsubo episode is consistent with recent data indicating that serotonin-norepinephrine reuptake inhibitor (SNRI) therapy is linked with lower short- and long-term mortality and fewer complications. This illustrates the possibility of having an early mental health intervention when it comes to the recovery process [30]. Observed reductions in smoking and increased early use of antidepressants/anxiolytics likely reflect a combination of physician-guided prescriptions and patient-initiated behavioral changes during the recovery phase. Early use of antidepressants and anxiolytics is consistent with guideline-recommended management for TCM patients with comorbid mood disorders, aiming to reduce stress-related autonomic activation and recurrence risk [31].

Limitations

Although the study examined key associations between ED and symptoms affecting the body’s automatic functions in TCM, it has several significant limitations. First, the cross-sectional design prevents the establishment of causal relationships or the assessment of the temporal sequence between ED and autonomic dysfunction. Second, data were collected using self-report questionnaires (DERS, COMPASS-31, HADS), which may be influenced by social desirability or recall biases. While participants were assured of anonymity and confidentiality to mitigate this bias, the lack of physiological validation for autonomic symptoms (e.g., heart rate variability) limits the objectivity of these measures. Third, convenience sampling from selected public and private hospitals in Pakistan may have influenced the demographic and clinical characteristics of participants, limiting the generalizability of the findings. In addition, the original English versions of the questionnaires have not been formally validated in the Pakistani population. Although bilingual interviewers assisted participants with limited English proficiency, cultural differences may still affect responses. Finally, an imbalance in gender distribution or the type of hospital (public vs. private) may further constrain generalizability to other populations.

Future directions

Future research could employ designs that follow individuals over time to investigate the ongoing relationship between ED and autonomic symptoms. Ideally, longitudinal studies should include follow-up periods of 6-12 months or longer to capture meaningful changes in TCM recurrence and symptom patterns. Recording patients’ feelings before, during, and after experiencing trouble controlling bodily activities could help determine whether emotional problems are the cause or effect of difficulties with physical control. It will also be vital to use electrocardiography, measure heart rate variations, and perform stress tests to make confident diagnoses and ensure the usefulness of research. Integrating wearable technology, such as heart rate variability monitors, could provide continuous autonomic monitoring for more precise physiological assessment. Another approach is interventional research, which involves analyzing the outcomes of psychological treatments, such as cognitive-behavioral therapy, mindfulness-based stress reduction, or emotion regulation, in patients with TCM. Future interventions could be personalized based on individual emotion regulation profiles rather than generic therapy. This analysis may reveal new methods to enhance their results. Conducting multicenter studies that involve diverse cultural and ethnic groups would provide a more comprehensive understanding of the role of culture in emotions and heart disease. Qualitative studies could explore cultural nuances in emotional expression that may influence responses to questionnaires. Lastly, with the help of functional imaging, scientists could observe the features that link the brain to the heart and develop a better understanding of how the condition arises. Combining neuroimaging with psychophysiological measures may provide a more comprehensive understanding of brain-heart interactions in TCM.

## Conclusions

We highlighted that ED was closely linked with autonomic dysfunction in TCM patients, even after controlling for anxiety and depression as measured by HADS scores (partial correlation: r = 0.283, p < 0.001, small-to-moderate effect size). This finding supports the adoption of a biopsychosocial approach to TCM, in which psychological interventions, particularly cognitive-behavioral therapy, mindfulness-based stress reduction, and emotion regulation training, are integrated into cardiac management. Such interventions may help reduce autonomic symptoms and enhance overall recovery. These results underscore the value of routine mental health screening and early referral to mental health services in cardiac care protocols. Although the cross-sectional design limits causal inference, the study provides a significant basis for future longitudinal and interventional research to assess the causal direction between ED and autonomic dysfunction and to refine clinical strategies for comprehensive patient management.

## Appendices

**Table 11:** Difficulties in Emotion Regulation Scale (DERS) Credits: Gratz & Roemer (2004) – Permission obtained for use in this study [14].

Items	Responses
I am clear about my feeling	-
I pay attention to how I feel	-
I experience my emotions as overwhelming and out of control	-
I have no idea how I am feeling	-
I have difficulty making sense out of my feelings	-
I am attentive to my feelings	-
I know exactly how I am feeling	-
I care about what I am feeling	-
I am confused about how I feel	-
When I’m upset, I acknowledge my emotions	-
When I’m upset, I become angry with myself for feeling that way	-
When I’m upset, I become embarrassed for feeling that way	-
When I’m upset, I have difficulty getting work done	-
When I’m upset, I become out of control	-
When I’m upset, I believe that I will remain that way for a long time	-
When I’m upset, I believe that I’ll end up feeling very depressed	-
When I’m upset, I believe that my feelings are valid and important	-
When I’m upset, I have difficulty focusing on other things	-
When I’m upset, I feel out of control	-
When I’m upset, I can still get things done	-
When I’m upset, I feel ashamed with myself for feeling that way	-
When I’m upset, I know that I can find a way to eventually feel better	-
When I’m upset, I feel like I am weak	-
When I’m upset, I feel like I can remain in control of my behaviours	-
When I’m upset, I feel guilty for feeling that way	-
When I’m upset, I have difficulty concentrating	-
When I’m upset, I have difficulty controlling my behaviours	-
When I’m upset, I believe that there is nothing I can do to make myself feel better	-
When I’m upset, I become irritated with myself for feeling that way	-
When I’m upset, I start to feel very bad about myself	-
When I’m upset, I believe that wallowing in it is all I can do	-
When I’m upset, I lose control over my behaviours	-
When I’m upset, I have difficulty thinking about anything else	-
When I’m upset I take time to figure out what I’m really feeling.	-
When I’m upset, it takes me a long time to feel better	-
When I’m upset, my emotions feel overwhelming	-

**Table 12:** Composite Autonomic Symptom Score (COMPASS-31) Credits: Sletten et al. (2012) – Permission obtained for use in this study [15].

Items	Responses
In the past year, have you ever felt faint, dizzy, “goofy”, or had difficulty thinking soon after standing up from a sitting or lying position?	-
When standing up, how frequently do you get these feelings or symptoms?	-
How would you rate the severity of these feelings or symptoms?	-
In the past year, have these feelings or symptoms that you have experienced	-
In the past year, have you ever noticed color changes in your skin, such as red, white, or purple?	-
What parts of your body are affected by these color changes? (Check all that apply)	-
Are these changes in your skin color	-
In the past 5 years, what changes, if any, have occurred in your general body sweating?	-
Do your eyes feel excessively dry?	-
Does your mouth feel excessively dry?	-
For the symptom of dry eyes or dry mouth that you have had for the longest period of time, is this symptom	-
In the past year, have you noticed any changes in how quickly you get full when eating a meal?	-
In the past year, have you felt excessively full or persistently full (bloated feeling) after a meal?	-
In the past year, have you vomited after a meal?	-
In the past year, have you had a cramping or colicky abdominal pain?	-
In the past year, have you had any bouts of diarrhea?	-
How frequently does this occur?	-
How severe are these bouts of diarrhea?	-
Are your bouts of diarrhea getting	-
In the past year, have you been constipated?	-
How frequently are you constipated?	-
How severe are these episodes of constipation?	-
Is your constipation getting	-
In the past year, have you ever lost control of your bladder function?	-
In the past year, have you had difficulty passing urine?	-
In the past year, have you had trouble completely emptying your bladder?	-
In the past year, without sunglasses or tinted glasses, has bright light bothered your eyes?	-
How severe is this sensitivity to bright light?	-
In the past year, have you had trouble focusing your eyes?	-
How severe is this focusing problem?	-
Is the most troublesome symptom with your eyes (i.e. sensitivity to bright light or trouble focusing) getting	-

**Table 13:** Hospital Anxiety and Depression Scale (HADS) Credits: Zigmond & Snaith (1983) – Permission obtained for use in this study [16].

Items	Responses
I feel tense or 'wound up'	-
I feel as if I am slowed down	-
I still enjoy the things I used to enjoy	-
I get a sort of frightened feeling like 'butterflies' in the stomach	-
I get a sort of frightened feeling as if something awful is about to happen	-
I have lost interest in my appearance	-
I can laugh and see the funny side of things	-
I feel restless as I have to be on the move	-
Worrying thoughts go through my mind	-
I look forward with enjoyment to things	-
I feel cheerful	-
I get sudden feelings of panic	-
I can sit at ease and feel relaxed	-
I can enjoy a good book or radio or TV program	-
